# Climate-driven shifts in algal-bacterial interaction of high-mountain lakes in two years spanning a decade

**DOI:** 10.1038/s41598-018-28543-2

**Published:** 2018-07-06

**Authors:** Juan Manuel González-Olalla, Juan Manuel Medina-Sánchez, Ismael L. Lozano, Manuel Villar-Argaiz, Presentación Carrillo

**Affiliations:** 1 Departamento de Ecología, Facultad de Ciencias, Universidad de Granada, 18071 Granada, Spain; 2 Instituto del Agua, Universidad de Granada, 18071 Granada, Spain

## Abstract

Algal-bacterial interactions include mutualism, commensalism, and predation. However, how multiple environmental conditions that regulate the strength and prevalence of a given interaction remains unclear. Here, we test the hypothesis that the prevailing algal-bacterial interaction shifted in two years (2005 *versus* 2015), due to increased temperature (T) and Saharan dust depositions in high-mountain lakes of Sierra Nevada (S Spain). Our results support the starting hypothesis that the nature of the prevailing algal-bacterial interaction shifted from a bacterivory control exerted by algae to commensalism, coinciding with a higher air and water T as well as the lower ratio sestonic nitrogen (N): phosphorous (P), related to greater aerosol inputs. Projected global change conditions in Mediterranean region could decline the functional diversity and alter the role of mixotrophy as a carbon (C) by-pass in the microbial food web, reducing the biomass-transfer efficiency up the web by increasing the number of trophic links.

## Introduction

Algae and bacteria numerically dominate the ocean and freshwater communities^1^, comprising the majority fraction of particulate organic carbon^2^. Hence, the interactions between algae and bacteria are crucial in aquatic environments as they control nutrient cycles and biomass production in the trophic web^3^. These relationships encompass commensalism^4^, predation^5^ and mutualism^6^, forming a continuum of the interaction modes^7^. In fact, several studies have reported the commensalistic interaction between the two metabolic groups^8,9^, where bacteria derive C for growth from the excreted organic carbon (EOC) provided by algae^1,10^. Thus, bacteria can consume up to 50% of the C fixed by phytoplankton^11^. In predation (i.e. bacterivory), bacteria supply C and nutrients (N, P or Fe) to mixotrophic algae^12,13^. Mutualism is established when bacteria, by decomposing organic matter, supply mineral nutrients^14^ or vitamins^15^ for algal growth^3^ while the algae supply C for bacterial growth. Furthermore, a complex mutualistic interaction develops when a dual control acts simultaneously through the bacterial dependence on EOC (i.e. a resource-based control), together with a predatory control by mixotrophic algae^16,17^.

The nature and strength of these interspecific interactions can change depending on environmental conditions^7^. Thus, nutrient input can alter the interaction in several ways. For example, moderated nutrient amendments can shift an algal–bacterial interaction from commensalism to competition depending on bacterial N:P ratio^18,19^, whereas high P enrichment (>30 µg P L^−1^) favours the development of strict autotrophic against mixotrophic algae, thereby reinforcing commensalistic interaction^17^. In addition, the strength of algal-bacterial interaction is regulated by T, due to a stimulatory effect on phytoplankton^20^, which increase EOC, supporting bacterial carbon demand and reinforcing the commensalistic relationship^21^. These findings contrast with the stronger effect of T on heterotrophic than autotrophic processes predicted by metabolic theory^22^ (although a recent study^23^ has questioned this theory), implying that, at the organism level, there is a shift toward heterotrophic metabolism of mixotrophic algae through increased bacterivory^24^. Therefore, the predominance of strict autotrophs or mixotrophs might encourage commensalistic *or* bacterivory interaction under warming, with the consequent effect on the regulation of the microbial food web.

Furthermore, warming can strength stratification in lakes, and therefore the exposure of microorganisms to ultraviolet radiation (UVR) in the upper layers of the water column^25^. It has widely been reported that UVR has a negative impact on several targets and processes (e.g. photosynthesis, and nutrient uptake, primary production (PP) and heterotrophic bacterial production (HBP))^26^. Nevertheless, it has also been demonstrated that planktonic organisms have a great capacity to acclimatize to high UVR in oligotrophic ecosystems by increasing the percentage EOC^27^, stimulating the growth of UVR-resistant bacteria^28^ or increasing the consumption of bacteria by mixotrophic algae^16,29^.

The joint effect of nutrient enrichment, T, and UVR on algal-bacterial interaction and microbial food web has been reported from experiments in single lakes^30–32^. However, little attention has been placed on the way in which these environmental factors alter the algal–bacterial relationship across systems over the long term. In this context, our study was conducted in high-mountain lakes distributed in an area of 86,200 ha in Sierra Nevada National Park, within the Mediterranean region. The effects of global change are accentuated in this region^33^, and even more so in high-elevation areas compared to the global average^34^, due to their greater ecological sensitivity to such change^35^. Particularly, the Mediterranean region is exposed to the increasingly nutrient-rich aerosol inputs from the Saharan Desert^36^, and Sierra Nevada is specifically receptive to Saharan dust due to its location and altitude^37^. In addition, the high elevation implies that the lakes are exposed to high-intensity UVR during the ice-free period, which may prolong over time because of rising T. These characteristics, together with their oligotrophic state and simple trophic web structure, make these lakes particularly sensitive indicators of the past and current global conditions and also serve as models to predict future changes because they register environmental change more directly^38^, being considered sentinels of global change^39^.

For these reasons, our objective was to assess whether the regulation of the algal-bacterial interaction has changed in 2015 respect to 2005, after a period in which Saharan dust transport to the Mediterranean basin and air T have increased, while chronic UVR levels remain high. Our hypothesis is that greater dust deposition to Sierra Nevada lakes and higher T have shifted the algal metabolism towards stricter autotrophy and higher PP, and therefore, we expect a reinforcement of the commensalistic interaction between algae and bacteria to the detriment of the predatory interaction.

## Results

### Remote sensing data

**Figure 1:**
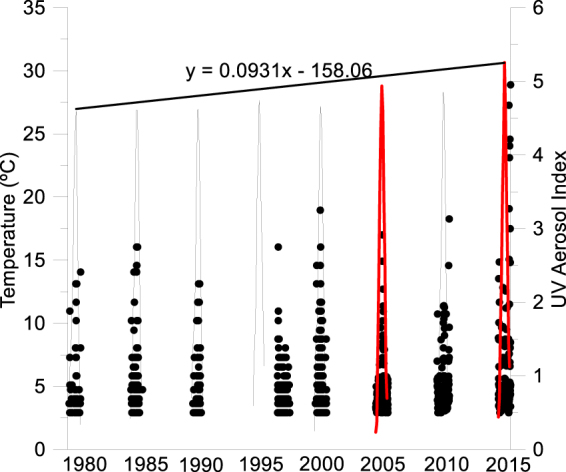
Environmental conditions (air Temperature (represented as lines) and UV aerosol index (represented as points)) for Sierra Nevada region (37°1′, −3°23′, 37°4′, −3°17′) for 1980 to 2015 period. Diagonal line represents the temperature trend during the ice-free period and the red lines the temperature of the two years studied.

**Figure 2:**
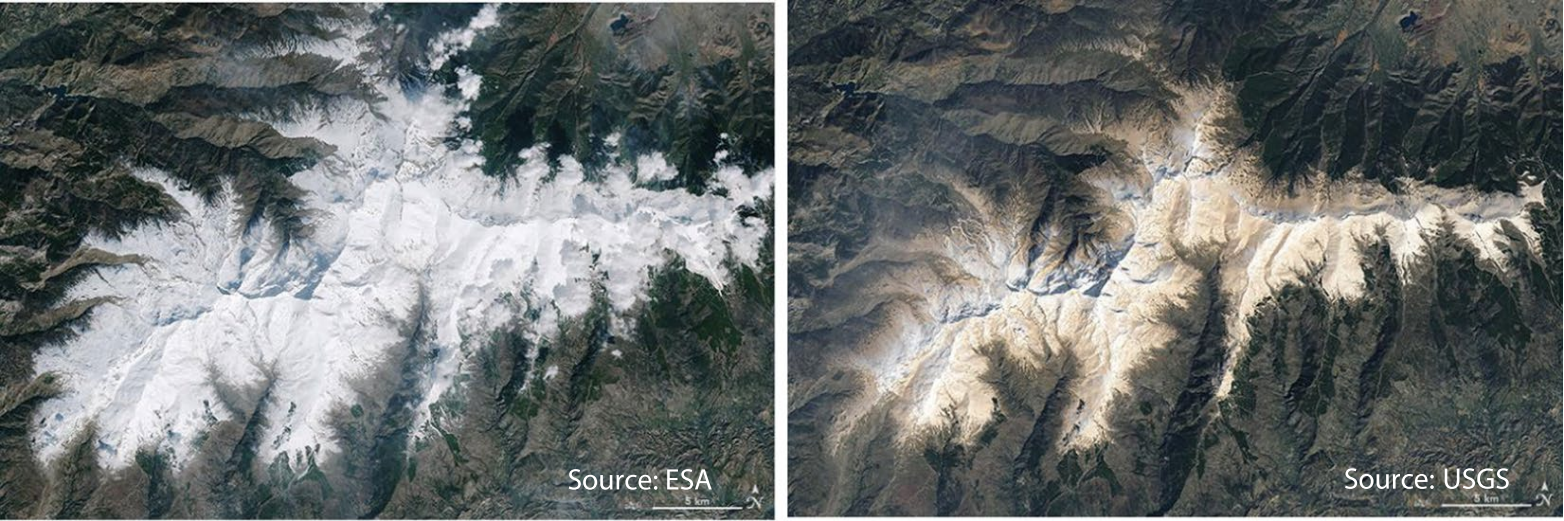
Aerial photography captured by the Sentinel 2-A (Left; courtesy of European Space Agency, ESA) and Landsat 8 (Right; courtesy of the U.S. Geological Survey) satellites corresponding to February 18 and February 27, 2017, after an intrusion of atmospheric dust over the Sierra Nevada National Park.

Environmental factors associated with global change have steadily varied over the last 35 years from the south-eastern Iberian Peninsula. While UVR striking the Sierra Nevada Mountains remained high during the summer period (300.25–306.47 W m^−2^; Supplementary Fig. S1), Saharan dust input and air T followed a positive trend during the 10 years covered by our metabolic measurements (Fig. 1). The UV aerosol index has increased progressively from the 1980s on, both in intensity and frequency (Fig. 1), accompanied by dust inputs during the winter of recent years, which is highly unusual in this region (Fig. 2). Specifically, during the last decade the aerosol index intensity has increased from 0.39 in 2005 to 0.44 in 2015, and the frequency of high-intensity events (aerosol index >1) has increased from 22 in 2005 to 49 in 2015. Also, the mean air T in summer has risen from 15.42 °C in 2005 to 16.45 °C in 2015.

### Abiotic and biotic changes in the lakes in 2005 and 2015

Diffuse attenuation coefficient for downward radiation (*kd*) showed low values of UVR (305 nm) and photosynthetically active radiation (PAR) throughout the water column, with the lakes Gabata, Larga, Caldera, and Yeguas exhibiting greater transparency to UVR (*kd* UVR_305_ = 0.31–0.92 m^−1^), while the lakes Chico de la Virgen, Grande de Rio Seco, and Alta de Río Seco reached the highest values (*kd* UVR_305_ = 4.04–7.5 m^−1^). The mean water-column T for the 10 lakes sampled in both years ranged between 15.5 °C in 2005 and 16.1 °C in 2015, and 60% of the lakes analysed had higher T in 2015, although without significant differences between the two years (*t*-test p > 0.05).

The trophic status of the lakes sampled in 2005^21^ and 2015 spanned oligo- to mesotrophy, as indicated by the total dissolved P, with values ranging from 0.049 to 0.483 μM of P in the lakes Larga and Aguas Verdes, respectively (Table 1). No significant differences appeared in total dissolved P concentration between the two years (*t*-test p > 0.05). However, the mean value of the seston N:P ratio for the 10 lakes sampled both years was 48.9, with 50.0% of the lakes showing values higher than 30 in 2005, whereas mean N:P ratio decreased to 18.75 with only 11.1% of the lakes showing higher values than 30 in 2015 (*t*-test p > 0.05). The mean concentrations of chlorophyll *a* (Chl *a*) and dissolved organic carbon did not significantly differ (*t*-test p > 0.05) between years (3.84 µg Chl *a* L^−1^ and 95.0 μM in 2005; 5.41 µg Chl *a* L^−1^ and 53.25 μM in 2015; Table 1).

**Table 1:** Characterization of high-mountain lakes of Sierra Nevada for 2005 and 2015.

	LAKES	*k*_*d* UVR 305_	*k*_*d* PAR_	Tª	TDP	TDN	DOC	Seston N:P	HBP	PP_P_	EOC	Bacterivory	Chl *a*	BA
		(m^−1^)	(m^−1^)	(°C)	(µM)	(µM)	(µM)		(µg C L^−1^ h^−1^)		(µg C L^−1^ h^−1^)		(µg L^−1^)	(cell ml^−1^ × 10^5^)
2005	Caballo	5.75	0.64	15	0.43	30.40	77	50.26	0.025	0.300	1.090	0.005	0.66	5.90
	Yeguas 1	0.61	0.2	15.50	0.10	7.10	89	227.11	0.008	2.651	0.530	0.005	7.77	2.40
	Yeguas 2	2.03	0.66	15.39	0.14	25.00	na	45.21	0.018	1.824	1.280	0.008	11.99	4.30
	Grande de la Virgen	1.19	0.45	11.80	0.13	9.10	65	32.55	0.021	0.943	1.860	0.019	0.54	15.70
	Chico de la Virgen	7.12	0.94	20.30	0.30	21.40	45	19.25	0.409	14.341	8.280	0.039	10.04	33.10
	Aguas Verdes	7.11	1.05	16.20	0.48	34.50	123	19.93	0.228	1.123	1.940	0.031	2.03	7.30
	Alta de Río Seco	4.07	2.66	15.50	0.33	23.20	115	13.39	0.052	0.155	1.480	0.009	0.43	2.40
	Grande de Río Seco	4.35	1.82	17.10	0.30	21.40	98	12.91	0.045	0.100	0.440	0.014	1.39	2.30
	Gabata	0.92	0.38	12.20	0.10	7.20	76	41.82	0.067	5.595	2.870	0.017	3.6	7.70
	Larga	0.37	0.2	16.10	0.12	8.30	85	60.67	0.009	0.189	0.240	0.004	0.25	3.50
	Caldera	0.38	0.18	15.73	0.19	13.80	87	36.42	0.017	0.352	3.280	0.002	1.86	6.50
	Caldereta	2.02	0.66	17.64	0.25	18	103	14.50	0.200	4.381	3.380	0.064	4.94	1
	Borreguil	3.74	0.82	14.50	0.28	19.60	140	18.48	0.263	0.189	1.230	0.002	0.69	1.40
	Hondera	3.49	1.6	16.40	0.26	18.70	150	26.43	0.378	5.595	2.760	0.072	11.85	8.50
2015	Yeguas 1	0.49	1.18	12.77	0.14	16.64	33.33	13.24	0.052	2.792	0.005	0.009	3.16	6.39
	Grande de la Virgen	1.24	0.35	11.46	0.16	19.50	29.46	na	0.021	0.192	0.753	0.001	1.68	1.40
	Chico de la Virgen	7.50	1.05	20.40	0.10	11.79	77.71	0.71	0.032	4.091	1.693	0.001	3.04	4.93
	Tajos Coloraos	1.63	0.42	17.76	0.07	16.29	31.98	38.75	0.035	0.060	0	0.012	2.03	2.43
	Borreguil	1.96	0.38	17.37	0.09	19.57	60.88	26.19	0.114	3.586	5.226	0.011	4.25	10.88
	Hondera	3.16	1.05	17.03	0.09	21.21	43.21	30.32	0.301	32.143	8.233	0.057	5.12	17.31
	Vacares	0.92	0.21	17.29	0.13	55.43	144.88	35.13	0.049	10.754	8.412	0.011	3.72	9.88
	Caldera	0.45	0.19	18.64	0.06	18.21	37.42	11.10	0.042	12.750	1.964	0.001	5.46	7.29
	Grande de Río Seco	4.66	0.57	18.68	0.18	17.43	81.15	18.39	0.239	28.404	3.966	0.006	6.46	40.62
	Alta de Río Seco	4.04	0.66	17.60	0.28	24.36	87.42	21.90	0.303	12.573	6.845	0.003	10.14	22.54
	Larga	0.68	0.27	19.51	0.05	9.29	56.13	22.95	0.032	5.171	2.008	0.008	2.15	1.05
	Gabata	0.31	0.36	8.08	0.20	26.57	25.79	23.95	0.003	0.030	0.052	0.0002	12.66	7.65
	Mosca	0.42	0.24	8.61	0.13	34.43	31.50	18.53	0.021	0.342	0.488	0.005	1.72	2.46

Units are given in brackets. TDP: Total dissolved phosphorous, TDN: Total dissolved nitrogen, DOC: Dissolved organic carbon, BA: Bacterial abundance.

**Figure 3:**
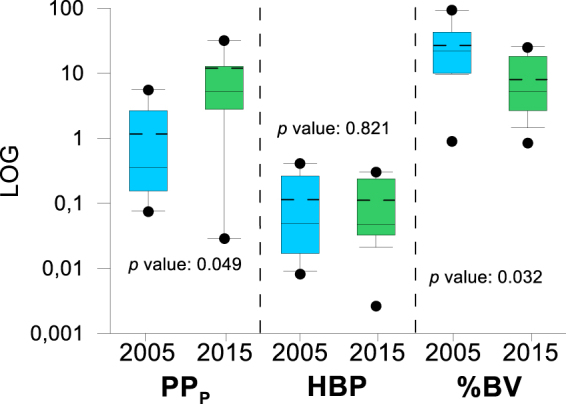
Box-Plot of particulate primary production (PP_P_), heterotrophic bacterial production (HBP) and % bacterivory (%BV) in Lakes of Sierra Nevada. Full lines and discontinuous lines inside the box represent the median and mean value, respectively. The boxes represent the upper and lower quartiles, while vertical lines indicate the 10^th^ and 90^th^ percentiles, and the points the 5^th^ and 95^th^ percentiles. 2005 and 2015 data were significantly different (paired *t-*test for dependent samples analysis) for PP_P_ and %BV with *p* value showed in the graph.

Regarding the functional variables, particulate primary production (PP_P_) registered a mean value of 1.71 µg C L^−1^ in 2005, significantly increasing in 2015 (10.85 µg C L^−1^; *t*-test, p = 0.049; Table 1 and Fig. 3). However, HBP mean values slightly decreased from 0.13 µg C L^−1^ h^−1^ in 2005 to 0.11 µg C L^−1^ h^−1^ in 2015, without significant differences between the two years (*t*-test p > 0.05; Table 1 and Fig. 3). The mean bacterivory rate (BV) was 18.48 ng C L^−1^ h^−1^ in 2005 and 9.71 ng C L^−1^ h^−1^ in 2015 (Table 1). The BV rates normalized by HBP (%BV) significantly decreased from 31.45% (2005) to 9.08% (2015 (*t*-test p = 0.032, Fig. 3).

### Factors controlling the algal-bacterial interaction in 2005

**Figure 4:**
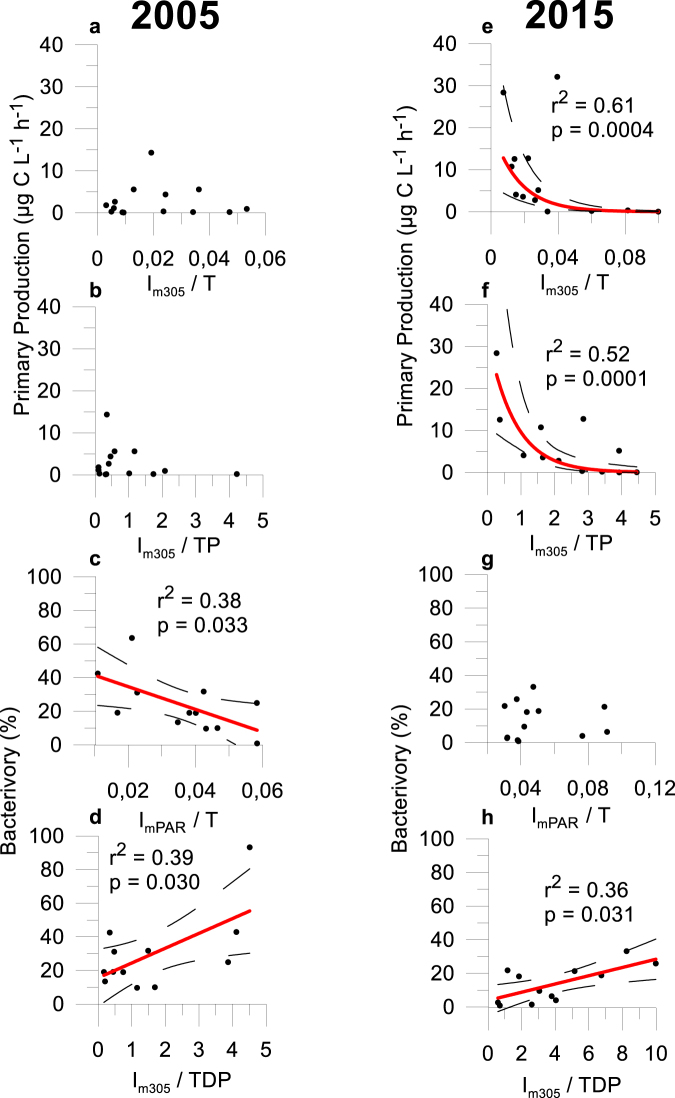
Response of PP_P_ and BV rate to abiotic factors (I_m305_:T, I_m305_:Total P (TP), I_mPAR_:T and I_m305_:Total dissolved P (TDP)) in Sierra Nevada Lakes in 2005 (left panel) and 2015 (right panel). Regression line, correlation coefficient (r^2^) and *p* value are represented only for years with a significant relationship.

The observed variability of PP_P_ of lakes during 2005 was explained by sestonic P (61.3% of variance; Table 2), according to the characteristic P limitation of the lakes (mean seston N:P ratio = 44.13). By contrast, water T, UV mean irradiance at 305 nm wavelength received throughout the water column (Im_305_), and the ratios between Im_305_ and T (Im_305_:T) or Im_305_ and total P (I_m305_:total P), failed to explain the PP_P_ during 2005 (Fig. 4a,b).

**Table 2:** Multiple stepwise regression for primary production (PP_P_), bacterivory (%BV) and bacterial production (HBP) in 2005 and 2015 (independent variables included in the table are only those which are statistically significant).

Dependent variable	Independent variable	n	Beta	Multiple R^2^	R^2^ exchange	*P*
*PP*_*P*_ *2005*	Sestonic P	14	0.783	0.613	0.613	0.003
*PP*_*P*_ *2015*	T	13	0.869	0.755	0.755	<0.001
*HBP 2005*	Chl *a*	14	0.899	0.440	0.440	0.013
	I_m305_		0.693	0.961	0.329	<0.001
	TDP		0.798	0.632	0.192	0.045
*HBP 2015*	TPP	13	0.580	0.706	0.706	<0.001
	TP		0.513	0.902	0.196	0.001
*%BV 2005*	Sestonic N:P	14	0.780	0.608	0.608	0.003
*%BV 2015*	Sestonic N:P	13	0.515	0.405	0.405	0.002
	Chl *a*		−0.561	0.795	0.389	<0.001
	I_m320_/TP		0.362	0.906	0.112	0.015

**Figure 5:**
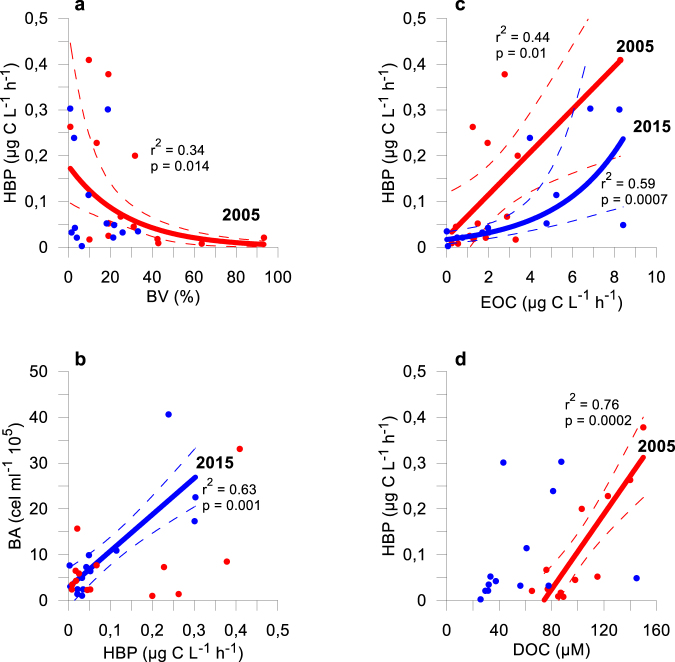
Relationship between Bacterial Production (HBP) and % bacterivory (%BV) (**a**), bacterial abundance (BA) (**b**), Excreted Organic Carbon (EOC) (**c**), and Dissolved Organic Carbon (DOC) (**d**). Data for 2005 and 2015 are represented in red and blue, respectively. Regression line, correlation coefficient (r^2^) and *p* value are represented only for years with a significant relationship.

HBP was explained by Chl *a* (44% of variance), I_m305_ (32.9%) and total dissolved P (19.2%; Table 2). The single regression assessing the C control on HBP showed a significant positive relation between EOC *vs*. HBP (Fig. 5c; r^2^ = 0.44; β-slope = 0.662; p < 0.05), and dissolved organic carbon *vs*. HPB (r^2^ = 0.76; β-slope = 0.873; p < 0.05); this appears to indicate that the C-control of HBP is exerted mainly by allochthonous C.

The %BV was explained by seston N:P (60.8%, p < 0.05, Table 2), suggesting that bacterivory was predominant in lakes with P impoverished seston. The single regression assessing the abiotic control (UVR, total dissolved P, and water T) on the %BV showed a positive relation between I_m305_: total dissolved P *vs*. %BV (r^2^ = 0.39; p < 0.05) but negative between I_mPAR_:T *vs*. %BV (r^2^ = 0.38; p < 0.05; Fig. 4c,d).

The high BV rates found for this year were reflected in a negative exponential relation (r^2^ = 0.34; p < 0.05) between HBP and %BV, suggesting a bacterivory control of algae on bacteria (Fig. 5a). Moreover, the lack of positive relationship between bacterial abundance and HBP (p > 0.05; Fig. 5b) is consistent with a top-down control.

### Factors controlling the algal-bacterial interaction in 2015

In 2015, water T explained the PP_P_ variance (75.5%, Table 2) and their relation was direct. Also, in contrast to 2005, the single regression assessing the abiotic control showed an exponential negative relation between I_m305_:T or I_m305_: total P and PP_P_ (Fig. 4e,f), according to a negative relation of I_m305_, but positive of P (Supplementary Fig. S2) or T on PP_P_ (Table 2).

Total primary production (TPP) and total P explained 70.6% and 19.6% of HBP variance, respectively (Table 2). The high relation between TPP and HBP is consistent with a significant direct C control between EOC and HBP, but not dissolved organic carbon (Fig. 5c,d). These findings support the contention that EOC rather than dissolved organic carbon controlled bacterial growth (bottom-up control; Fig. 5c,d), suggesting a reinforcement of the algal-bacterial commensalism in 2015.

Sestonic N:P ratio and I_m320_: total P were positively related to %BV, explaining 40.5% and 11.2% of %BV variance, respectively (Table 2, see also Fig. 4h), and Chl *a* was negatively related to %BV, explaining 38.9% of %BV variance (Table 2). These findings indicate that greater P availability among lakes simultaneously stimulated the Chl *a* concentration and weakened bacterivory.

The low %BV values found in 2015 (Fig. 3) together to the absence of bacterivory control between HBP and %BV (Fig. 5a) and a significant positive relationship (r^2^ = 0.63; p < 0.05) between bacterial abundance and HBP (Fig. 5b), consistently suggest a weak consumption of bacteria by the algae.

## Discussion

**Figure 6:**
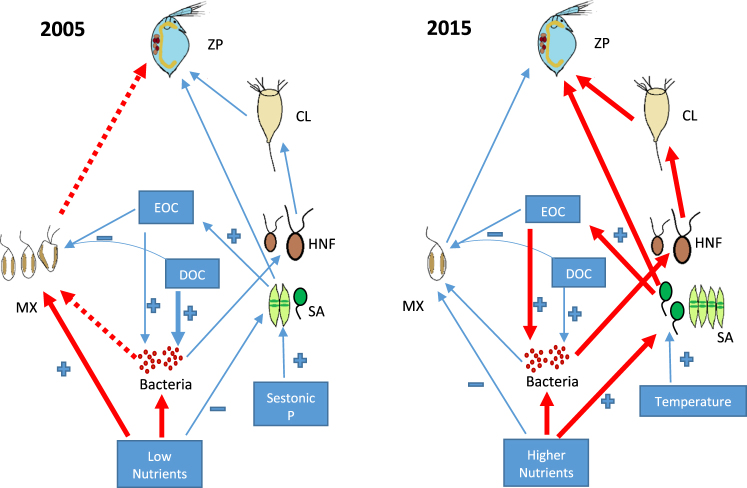
Scheme of the functional regulation of the trophic web in high mountain lakes in 2005 and 2015. Red and blue lines represent the type of interaction that is reinforced and diminished, respectively. Red dashed lines represent the C-bypass. ZP: zooplankton, CL: ciliates, HNF: heterotrophic nanoflagellates, SA: strict autotrophs, MX: mixotrophs, EOC: Excreted Organic Carbon, DOC: Dissolved Organic Carbon.

The main finding of our study was a shift in the nature of the interaction between algae and bacteria in high-mountain lakes of Sierra Nevada in 2005 and 2015, with a consistent and greater predominance of autotrophic metabolism in 2015 supported by an increase in the PP_P_, and with the reinforcement of commensalistic algal-bacterial interaction against the weakening of bacterivory control (Fig. 6). This supports our hypothesis and highlights that temperature increase and a dustier world^40^ like observed in 2015, can alter the functioning of high mountain lakes where BV rates strongly regulate the dynamics and structure of the microbial community.

The BV rate was altered in the two years of our analysis. In 2005, the predatory control by bacterivores rose to some 30%, this becoming a characteristic feature of the high-mountain lakes of Sierra Nevada. This feature is supported by predominance of mixotrophic organisms in Sierra Nevada lakes^4,16^. Field studies have revealed that mixotrophic algae are often numerically dominant in freshwater systems and can exert greater grazing impact on the bacterial community than can heterotrophic phagotrophs^29,41^. Concretely, in 2005 the bacterivory of mixotrophs could constitute an advantageous nutritional mode against strict metabolisms under the typically low P concentration of Sierra Nevada Lakes (see Table 2)^17,30^ as has also been observed in other lakes with low P values^42,43^. In addition, the fact that %BV was positively correlated to I_m305_:Total dissolved P ratio supports the idea that mixotrophy is an adaptive strategy to low P levels and also high UVR levels^29,44^. The great adaptation of mixotrophs to high light intensity could be related with its capacity to grow as photoheterotrophs^45^, reducing the costs of investment in photoprotective mechanisms for photosystem II. Several studies have demonstrated increased grazing rates by mixotrophs in the epilimnion under high irradiance levels^16,46^. The reported adaptation of mixotrophs to low nutrient conditions^5,44^ is supported by loss of mixotrophs after a strong P-pulse as has been demonstrated experimentally in Sierra Nevada lakes^17,30^, in Andean lakes^29^, in tropical and temperate lakes^47^, and in the laboratory^48^ as well as through studies of gradients of increasing trophic state^49^.

In fact, in 2015, the %BV fell as much as 12.85% (Fig. 3) as the dust input intensified (Fig. 1), the sestonic N:P ratio diminished (Table 1), and concomitantly mixotrophs represented only 19.3% of the algae community as opposed to 61% of strict autotrophs in the ensemble of lakes (Supplementary Fig. S3). The %BV reduction implied a weakening in bacterivory control with respect to 2005 (Fig. 5a), which was corroborated by a bacterial abundance:HBP coupling in 2015 (Fig. 5b). In this sense, in a coastal upwelling, it has been reported that when bacterial mortality by predation is low, all HBP can be converted into BA, and conversely, the absence of a bacterial abundance:HBP coupling is indicative of a strong bacterivory control^50^.

Contrary to 2005, in 2015 the predominant algal-bacterial interaction was commensalism, led by higher PP_P_ (and Chl *a*) (Fig. 3). The PP_P_ was triggered mainly by water T (Table 2) concomitantly with an increasing trend in aerosol dust intensity and air T on Sierra Nevada from 1980 to 2015 (Fig. 1). It is known that P, Fe, and other limiting nutrients contained in aerosol dust affect the phytoplankton physiology^51^ and enhance PP_P_ of lakes^17^ and coast^52^. The greater aerosol dust inputs to Sierra Nevada during the recent years of our study translated into a lower mean N:P ratio in 2015 and a lower percentage of lakes with a N:P ratio higher than 30 (see Results and Table 1), indicating P incorporation into seston, as also observed in previous studies^19^. The positive effect of nutrient-rich dust input in autotrophy could be enhanced by a stimulatory effect of T on phytoplankton photosynthetic yield^53^ and an attenuation of UVR inhibition on autotrophs^54^. Furthermore, rising T favoured the dominance of strictly autotrophic phytoplankton taxa, such as Chlorophyta^55^.

Supporting the advantage of strict autotrophy after greater aerosol inputs and higher water T, we observed an EOC increase proposed as a protective mechanism to prevent the photosystem damage under high light irradiance^56,57^. Furthermore, the EOC increase determined a reinforcement of commensalistic algal-bacterial interaction, since EOC was the main C source regulating HBP in 2015 whereas dissolved organic carbon was the source in 2005 (Fig. 5c,d and Fig. 6). In agreement with our results, an experiment in marine waters^58^ showed that the direct C coupling between bacteria and phytoplankton exudates was greatest when the nutrient concentration was higher and grazing was lower, coinciding with a predominance of small autotrophic cells as the main components of the phytoplankton community.

## Implications

The high UVR-adaptation of the algae inhabiting high-mountain lakes together with the rising water T and greater nutrient inputs linked to dust deposition that occurred in 2015 shifted the predominant algal-bacterial interaction from bacterivory to comensalism^59^. While we cannot establish a consolidated trend from our study, it is true that the projections of the climatic change for the present century could be more similar to those conditions that occurred in 2015. Thus, the loss of a functional trait such as mixotrophy in high-mountain lakes could deteriorate the functional biodiversity^29,60^ and biogeochemistry of these remote and fragile ecosystems, by altering processes such as C and nutrient cycles due to the disconnection between the microbial loop and higher trophic levels. Thus, the decrease in mixotrophs is expected to weaken the C bypass towards the grazing chain of the lakes^29,46^, promoting the development of heterotrophic microbial food web and diminishing the energy-transfer efficiency due to a greater number of links (Fig. 6)^16,61^.

## Material and Methods

### Remote sensing

The remote-sensing data for the Sierra Nevada area were gathered from 1980 to 2015. Daily data of the area-average aerosol index, air T, and surface UVR fluxes in this region were downloaded from Giovanni v. 4.18.3^62^. Aerosol index data were taken from the Total Ozone Mapping Spectrometer (TOMS) Nimbus-7 (March 21, 1979 – May 5, 1993), TOMS Earth Probe (July 22, 1996 – June 28, 2005) and the Ozone Monitoring Instrument (OMI) (June 29, 2005 – December 31, 2015) satellites (data from 1993 to 1996 are not available), while T and surface UVR-flux data came from the MERRA and MERRA-2 model, respectively. Aerosol index intensity was calculated as the mean value of aerosol index for each entire year, whereas the frequency of high load aerosol index events was calculated as the number of days per year with aerosol index >1.

### Study area

Our study was conducted in 14 lakes in 2005 and 13 in 2015, which 10 lakes were common between the two years. Some of the lakes could not be sampled in 2015 due to the severe hydric stress to which are subjected, affected by the Mediterranean climate. All lakes are located above the tree line in the Sierra Nevada Mountains (36.8559–37.8159 N, 2.8319–3.8409 W), in the southern Iberian Peninsula (Supplementary Fig. S4). Sampling was conducted during the ice-free period (mid-July to mid-August) of each year.

These lakes are oligo- and mesotrophic (Chl *a* range from 0.25 to 12.66 µg L^−1^). The external inputs of mineral nutrient occur mainly during thaws, and are associated with sporadic events of Saharan dust deposition^63,64^ containing high P levels, with a mean molar total N:total P ratio in total dust deposition ranging from 10 to 50^37^. The lakes are characterized by their small size (<3 ha), high transparency, shallow water column (<10 m maximum depth) and by the simplicity of the pelagic fishless community, with a low abundance of heterotrophic nanoflagellates and ciliates, and the presence or dominance of mixotrophic protists in the trophic web^4^.

### Field sampling

From each lake, an integrated sample representative of the mixing layer of the water column was collected using 10 L-Niskin Bottle and used for biological and chemical analyses. The samples were then filtered through a 45-µm pore-size mesh to remove zooplankton, and subsamples for each variable analysed were taken in triplicate (see below).

Morphometric and physical measurements were also performed for characterizing each lake (see below).

## Physical parameters

### Temperature and light measurement

Vertical profiles of radiation and T of water column were determined at noon using a submersible radiometer BIC (Biospherical Instruments Inc., CA, USA) that registered measurements of downwelling irradiance at wavelengths representative of the different regions of the solar spectrum (305, 320, and 380 nm and full PAR [400–700 nm]). The *k*_d_ were determined from the slope of the linear regression of the natural logarithm of downwelling irradiance *vs*. depth for each wavelength.

I_m_ for PAR and UVR was calculated as in equation (1):

1 $${{\rm{I}}}_{{\rm{m}}}=[{{\rm{I}}}_{0}\times [1-{{\rm{e}}}^{(-{k}_{{\rm{d}}}\times {\rm{z}})}]]\times {[{k}_{{\rm{d}}}\times {\rm{z}}]}^{-1}$$

where I_0_ is the mean incident surface irradiance; *k*_d_ is the mean attenuation coefficient for UVR at 305, 320, 380 nm and PAR; and z is the maximum depth for each lake.

### Chemical parameters

The concentrations of total P and total dissolved P were determined in 25-ml aliquots after digestion with a mixture of potassium persulphate and boric acid at 120 °C for 30 min^65^. Total N and total dissolved N samples were also digested with persulfate and measured as nitrate following the ultraviolet spectrophotometric screening method^65^. For determination of total dissolved N and total dissolved P, the water samples were previously filtered at low pressure (<100 mmHg) using glass-fibre filters (0.7 µm pore-size, Whatman GF/F). To determine sestonic C, N, and P, water samples (0.5 L for C/N and 0.5 L for P) were filtered through precombusted (1 h at 550 °C) glass-fibre filters of 1-µm pore size (Whatman GF/B) at low pressure (<100 mm Hg). The filters were immediately frozen at −20 °C, and the C and N analyses were performed using a Perkin-Elmer 2400 elemental analyser. The sestonic P was determined with the same method as that described for total P.

For dissolved organic carbon determination, samples were filtered through pre-combusted (2 h at500 °C) glass fibre filters (Whatman GF/F) and acidified with HCl 1 N. The analyses were carried out in a total organic carbon analyzer (TOC-VCSH/CSN Shimadzu).

## Biological parameters

### Chlorophyll-a concentration

Chl-*a* concentrations were determined by fluorometric techniques^66^. The samples were filtered onto a Whatman GF/F filter (0.7 µm pore size) and the photosynthetic pigments extracted in 5 ml of absolute methanol at 4 °C in darkness.

Samples were measured at an excitation wavelength of 460 nm and emission at 670 nm, with a fluorometer LS55 Luminescence Spectrometer (Perkin-Elmer, Boston, MA, USA). Previously, a calibration curve was made with pure chlorophyll spinach extract to transform fluorescence values in chlorophyll concentration.

### Primary production

Following the ^14^C method proposed by Steeman-Nielsen^67^, 140-ml samples from each lake were collected for PP measurements. Four 35-mL Teflon flasks for each treatment (three clear and one dark as control) were added with 9.25 MBq of NaH^14^CO_3_ (specific activity: 310.8 MBq mmol-1, DHI Water and Environment, Germany). The flasks were incubated for 4 h around noon, and the laboratory procedure was based on Carrillo *et al*.^56^. Thus, 4-ml aliquots were taken before filtration to measure the TPP produced, and 35-ml aliquots were filtered, retaining the organic carbon particles in Nuclepore filters of 1-µm pore size to determine the PP_P_. From the filtrates (<1 μm), 4 ml were used for measuring the EOC.

Filters and filtrates were placed in 5- and 20-mL scintillation vials, respectively, and acidified with 100 µL of 1 N HCl in order to remove DI^14^C. Vials were then kept open during 24 h in an aeration hood following the recommendations of Lignell^68^. Then, the vials were filled with scintillation cocktail (Ecoscint A) and counted using a scintillation counter (Beckman LS 6000TA) equipped with autocalibration.

The sum of EOC and PP_P_ corresponds with the TPP. The % EOC was calculated as in equation (2):

2 $$ \% {\rm{EOC}}=\frac{{EOC}}{{TPP}}\times 100$$

### Abundance/biomass of planktonic organisms

Abundance and biomass of algae, heterotrophic nanoflagellates, ciliates, and nanoplankton were counted at 400x and 1000x magnification under an inverted microscope (Carl Zeiss AX10, LCC, USA).

For the quantification of autotrophic picoplankton and bacteria^69^, several 1.5 mL aliquots from each sample were fixed with paraformaldehyde (1% final concentration) and immediately stored at −180 °C until further processing. Autotrophic picoplankton abundance was quantified based on Chl *a* auto-fluorescence, whereas total picoplankton abundance was quantified after other 1.5-mL fixed aliquots were stained with SYBR Green I DNA (Sigma-Aldrich) 1:5000 final dilution of initial stock. Absolute cell-abundance values were determined using a Becton Dickinson FACScan flow cytometer (Oxford, UK) and Yellow-green-l µm beads (Fluoresbrite Microparticles, Polysciences, Warrington, PA, USA).

### Heterotrophic bacterial production

HBP was determined as incorporation of 3H-thymidine (specific activity = 52 Ci mmol^−1^, Amersham Pharmacia) into the bacterial DNA. Briefly, 3H-thymidine was added to sets of six (four replicates plus two blanks per lake) sterile microcentrifuge tubes filled with 1.5 mL of sample to a final (saturating) concentration of 25 nM. The vials were then incubated at *in situ* T in darkness for 1 h. After incubation, the incorporation of 3H-thymidine was stopped by adding trichloroacetic acid (TCA, 5% final concentration). Likewise, blanks were TCA-killed before the radiotracer was added. After the cold 5% TCA extraction, the precipitate was collected by centrifugation at 16,000 g for 10 min, rinsed (and centrifuged) twice with 5% TCA, and measured in a scintillation counter equipped with autocalibration (Beckman LS 6000 TA). The conversion factor 1.5 × 10^18^ cell mol^−1^ was used to estimate the number of bacteria produced per mole of incorporated ^3^H-thymidine^70^. The factor 20 fg C cell^−1^ was applied to convert bacterial production into C^71^.

### Bacterivory

BV was determined from the amount of bacteria traced with ^3^H-thymidine that were captured by algae following the method proposed by Medina-Sánchez^16^. For determining BV, sets of four Teflon bottles (three replicates and one blank per lake) were filled with 25 ml of water samples each, radiotraced with ^3^H-thymidine to a final concentration of 25 nM, and incubated for 1 h in full sunlight. After incubation, the bacterivory was stopped by adding neutralized formaldehyde (0.75% w/v final concentration). Likewise, blanks were formaldehyde-killed before the incubation with the radiotracer. Aliquots of 1.5 ml were obtained from each replicate for determining total activity in the sample. The remaining sample volume (23.5 ml) was filtered through 1 µm pore-size cellulose nitrate filter (Sartorius). Aliquots of 1.5 ml were taken from the filtrate to determine the residual activity, while the activity registered on the filter served to determine BV. The filters were dissolved using acetone 100% and then subjected to centrifugation at 16,000 *g* for 10 min at 4 °C. Supernatant acetone was removed, and the pellet was re-suspended with 5% TCA (1.5 ml). The subsequent procedure was similar to HBP. %BV was calculated as in equation (3):

3 $$ \% {\rm{BV}}=\frac{\,{\rm{Bacterivory}}\,}{{\rm{HBP}}}\times 100$$

where bacterivory was determined from the bacterial incorporation rate by fraction >1 µm retained on the filter. Bacterivory values were converted to cell number and bacterial C captured using the same conversion factors as for HBP determination.

### Statistical analysis

Paired *t-*test for dependent samples analysis was carried out with the 10 lakes that were sampled both years (2005 and 2015) to determine significant differences in Chl-*a*, PP_P_, HBP, %BV, sestonic N:P ratio, dissolved organic carbon and T between 2005 and 2015. The homogeneity of the variances was verified by Levene’s and/or the Brown and Forsythe test.

SMR were carried out with all the lakes sampled each year (14 and 13 lakes in 2005 and 2015, respectively) to quantify the influence of biotic and abiotic factors (I_m305_, I_m320_, I_m380_, I_mPAR_, total dissolved P, total P, total dissolved N, total N, T, dissolved organic carbon, EOC, TPP, Chl *a*, I_m_:total dissolved P, I_m_: total P, I_m_: total dissolved N, I_m_:T, bacterial abundance) on PP_P_, HBP and %BV in 2005 and 2015. Linearity and multiorthogonality among independent variables were verified by previous correlation analysis and controlled by specifying 0.6 as the minimum acceptable tolerance. Because some factors co-vary with those included by SMR, single regression analyses were performed to assess strength of: i) the C-based control (EOC or dissolved organic carbon) on HBP; ii) the abiotic control (T, UVR, I_m305_:T, I_mPAR_:T, I_m305_:Total P, I_m305_: total dissolved P) on PP_P_ and %BV; iii) the predatory control (%BV or bacterial abundance) on HBP, to evaluate changes in the algal-bacterial interaction. The normal distribution of residues in all regressions was checked by Kolmogorov-Smirnov tests (Statsoft 1997).

### Data availability

The datasets generated during and/or analysed during the current study are available from the corresponding author (jmolalla@ugr.es).

## Electronic supplementary material

Supplementary information
