# Supplementary material for: Climate-driven shifts in algal-bacterial interaction of high-mountain lakes in two years spanning a decade
Source: Sci Rep. 2018 Jul 6;8:10278. doi: 10.1038/s41598-018-28543-2 (PMC6035198; doi:10.1038/s41598-018-28543-2)
Supplement: Supplementary file 1 — Supplementary information [file 41598_2018_28543_MOESM1_ESM.pdf]

Scientific Reports

Supplementary information for

Climate-driven shifts in algal-bacterial interaction of high-mountain lakes in two years spanning a decade

Juan Manuel González-Olalla*[1], Juan Manuel Medina-Sánchez[1], Ismael L. Lozano[1], Manuel Villar-Argáiz[1] & Presentación Carrillo[2]

[1]Departamento de Ecología, Facultad de Ciencias, Universidad de Granada, 18071 Granada, Spain

[2]Instituto del Agua, Universidad de Granada, 18071 Granada, Spain

*Email: jmolalla@ugr.es

Contents of this file

Figures S1 to S4

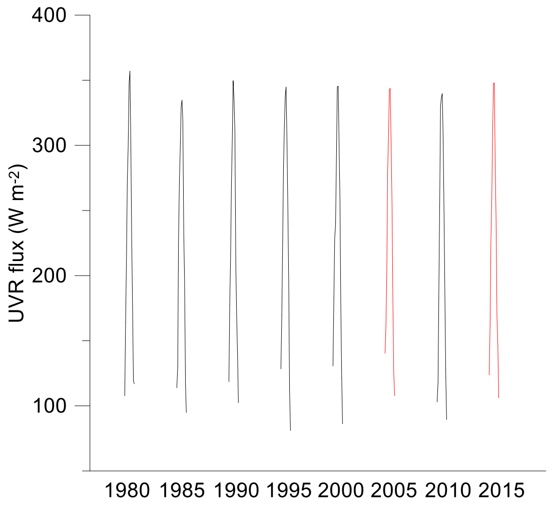

Figure S1. UVR flux incising on Sierra Nevada region (37º1', -3º23', 37º4', -3º17') for the period 1980 to 2015.

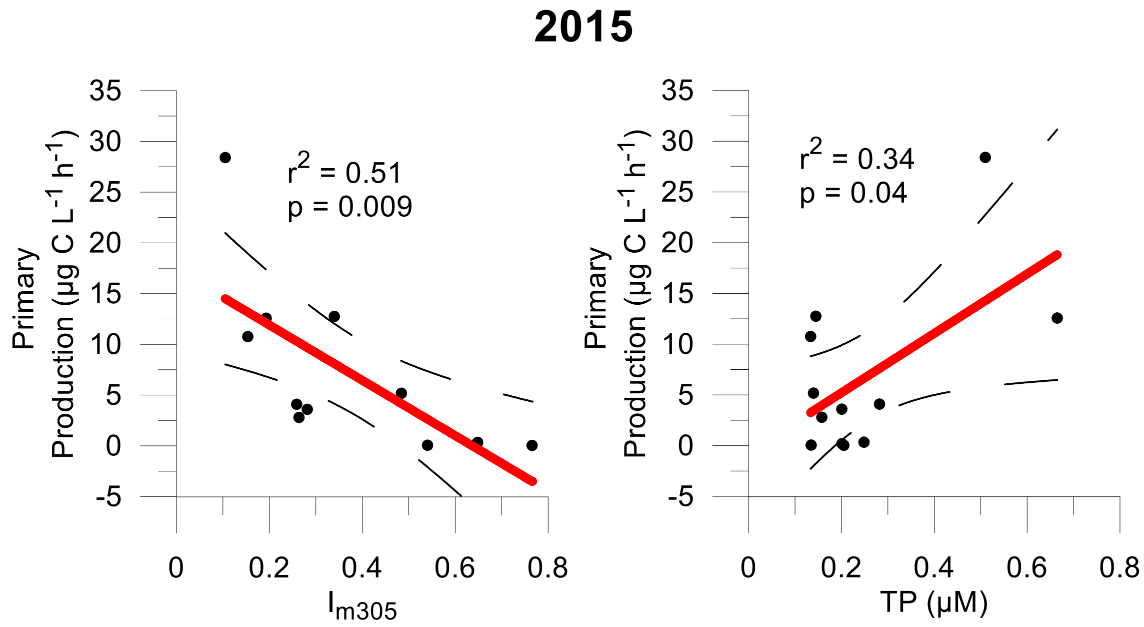

Figure S2. Response of PP_P to abiotic factors (I_{m305} and TP) in Sierra Nevada Lakes in 2015. Regression line, correlation coefficient (r^2) and *p* value are represented.

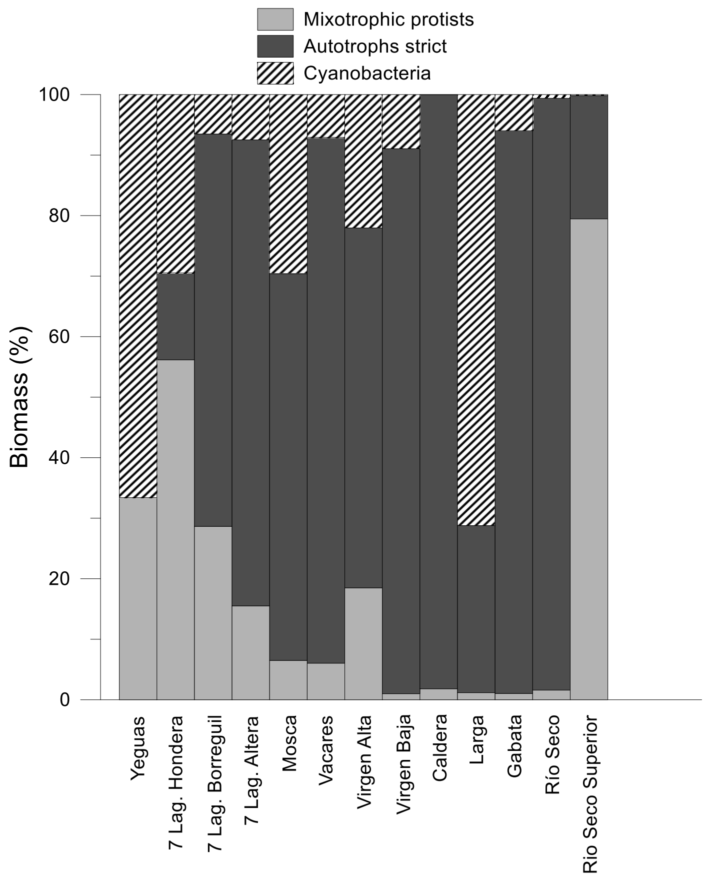

Figure S3. Biomass composition of Sierra Nevada lakes in July 2015.

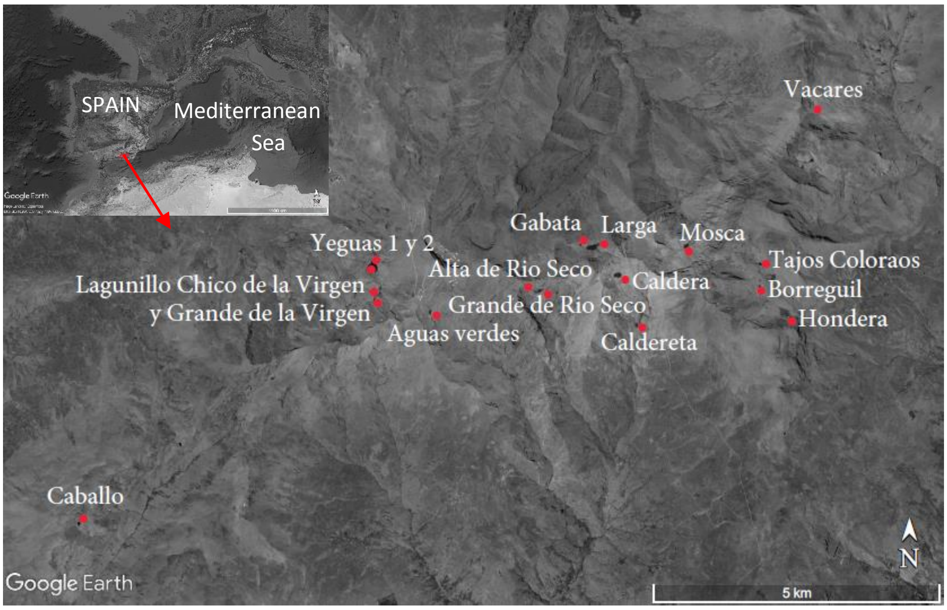

Figure S4. Location map of 17 Lakes of Sierra Nevada (southern Spain).
